# Criterion validity for step counting in four consumer-grade physical activity monitors among older adults with and without rollators

**DOI:** 10.1186/s11556-019-0235-0

**Published:** 2020-01-03

**Authors:** Rasmus Tolstrup Larsen, Christoffer Brun Korfitsen, Carsten Bogh Juhl, Henning Boje Andersen, Henning Langberg, Jan Christensen

**Affiliations:** 1 Department of Public Health, Faculty of Health and Medical Sciences, CopenRehab, Section of Social Medicine, University of Copenhagen, Gothersgade 160, 3rd floor, 1123 Copenhagen K, Denmark; 2 Research Unit of Musculoskeletal Function and Physiotherapy, Institute of Sports Science and Clinical Biomechanics, Faculty of Health Sciences, University of Southern Denmark, Odense, Denmark; 3 Department of Physiotherapy and Occupational Therapy, Copenhagen University Hospital, Herlev and Gentofte, Hellerup, Denmark; 4 Technical University of Denmark, DTU Management Engineering Inst, Diplomvej 372, 2800 Lyngby, Denmark; 5 Department of Occupational- and Physiotherapy, Copenhagen University Hospital, Rigshospitalet, Copenhagen, Denmark; 6 National Centre for Rehabilitation and Palliative Care, University of Southern Denmark and Odense University Hospital, Nyborg, Denmark

**Keywords:** Validity, Physical activity monitors, Walking, Technology

## Abstract

**Background:**

Few studies have investigated the measurement properties of consumer-grade physical activity monitors (PAMs) in older adults. Therefore, we investigated the criterion validity of consumer-grade PAMs in older adults and whether the measurement properties differed between older adults with and without rollators and whether worn on the hip or at the wrist.

**Methods:**

Consumer-grade PAMs were eligible for inclusion in this study if they: 1) could be fastened at the hip as well as on the wrist, 2) were simple in function and design and thus easy to use for participants with minimal technical skills, 3) included step-counting as outcome measure and 4) were powered by a button cell battery. Participants performed self-paced walking for six minutes while two physiotherapists counted their steps with a click-counter. The average of the two counts was used as criterion. The participants wore 16 monitors, four located bilaterally on both hips and wrists. Our prior expectation was that all monitors would have at least moderate criterion validity for all participants, good criterion validity for participants walking without a rollator and poor criterion validity for participants walking with a rollator.

**Results:**

Four physical activity monitors were included in this study; Misfit Shine, Nokia GO, Jawbone UP Move and Garmin Vivofit 3. A total of 103 older adults participated.

Nokia GO was excluded from this study due to technical issues. Therefore, we present results on the frequency of data loss, ICC (1, 2) and percentage measurement error for Misfit Shine, Garmin Vivofit 3 and Jawbone UP Move located on four different positions.

**Conclusions:**

The hip-worn PAMs did not differ significantly in terms of measurement error or criterion validity. Wrist-worn monitors cannot adequately measure number of steps in a population of older adults using rollators. The hip-worn PAMs were superior to wrist-worn PAMs among older adults with and without rollators.

## Background

Functional decline is related to aging. Still, older adults who engage in exercise or physical activity regularly can, to some extent, maintain their physical function, have lower all-cause mortality, are less disabled, and have a lower prevalence of several non-communicable diseases [1–4]. Walking is the favourite activity among community-dwelling cognitively-intact older adults [5]. Furthermore, walking programmes have in several systematic reviews been shown to be effective in increasing physical activity in the short term in older adults [6]. However, to ensure long-lasting effects and adherence of walking programs, they should be individualised and based on behavioural theories, as well as include goals to maintain acceptable levels of PA [6].

To use goal setting in the individualisation of walking programs, individual feedback on PA is crucial. The consumer-grade physical activity monitors (PAMs) hold the potential of being a facilitator for increased PA as they provide timed feedback, notifications and can be adjusted with individual goals [7]. For these reasons, PAMs are now frequently used with good effect to increase physical activity in older adults [8, 9]. However, before using consumer-grade PAMs in clinical research, the measurement properties, including criterion validity in particular, of specific PAMs should be evaluated [10]. Measurement properties for specific PAMs may differ between different populations of older adults. Thus, it has been shown that adults suffering from knee pain or those who depend on a walker have different gait characteristics compared with normal older adults [11]. Within the population of older adults, a large heterogeneity exists in gait speed, stride length, joint movement, and use of assistive devices, all of which have been found to affect the validity of PAMs [11, 12].

Furthermore, consumer-grade PAMs differ from research-grade PAMs because the algorithms for step detection cannot be modified and thus the definition of a step might differ between PAMs. Hence, there is no transparency in the use of algorithms. Besides, most modern consumer-grade PAMs are designed to be worn on the wrist as watches, which might lead to inaccurate measurement as hip-worn PAMs have been reported to outperform wrist-worn PAMs for step accuracy [13].

To our knowledge, few studies have investigated the measurement properties of consumer-grade PAMs in older adults, and none of these has studied the measurement properties of a given PAM model worn on the hip and wrist [12, 14–20]. Therefore, the present study aimed to investigate (a) the criterion validity of four consumer-grade PAMs in older adults performing a self-paced indoor walking test and (b) whether the measurement properties of the PAMs differed between older adults with and without rollators and comparing wrist-worn and hip-worn positions.

## Methods

### Participants

We included older adults from five community activity centres in the municipality of Copenhagen, Denmark. The participants were recruited at the ‘morning meet-up’ where our research team presented the study. Participants were eligible if they were 65 years or above, community-dwelling, living at home and able to walk independently with or without a rollator or cane. Mild and more severe cognitive impairment was an exclusion criterion, since participants had to be able to understand the study aims and fill out themselves the baseline questions.

### Ethics

Oral and written information was given before participants gave informed consent to participate. The study was approved by the Danish Ethics Committee (Journal nr.:H-17033310).

### Physical activity monitors

As we could not investigate all available PAMs, we chose those who were most relevant for older adults and those who allowed us to investigate whether the placement of the specific PAM affected the validity. Thus, consumer-grade PAMs were eligible for inclusion in this study if they: 1) could be fastened at the hip as well as on the wrist, 2) were simple in function and design and requiring no technical skills to be operated, 3) included step-counting as the outcome measure and 4) powered by a button cell battery providing a battery life for more than three months. If the included PAMs did not have a display, they were paired with an iPod Touch 5th generation, model A1421, operating with iOS 9.3.5. We performed pilot testing of all the eligible consumer-grade PAMs within the research team before conducting the present study.

### Procedures and measures

Participants were included between March and June 2018. In the five activity centres, participants were asked to perform self-paced walking for six minutes. To secure the external validity of our results, we asked the participants were asked to walk at their normal gait speed, instead of a maximal walking test.

An unobstructed 15- or 30-m flat track was used for testing, at each end a cone was positioned indicating where participants should make a 180-degree turn. The participants decided themselves whether they performed right or left turns. If the participants were interrupted during the testing or became tired, they were allowed to rest standing or sitting and the time was stopped until they continued. A chair was provided upon request. The participants received no verbal feedback on gait speed from the testers. The participants were fitted with 16 PAMs (four models located bilaterally on both hips and wrists). The hip-worn monitors were fitted to the belt of the participant or to front pocket sewing, the wrist-worn monitors were fitted with the rubber straps provided, and in both cases testers assisted with fitting.

The order of the PAMs was changed between every participant to ensure a balanced order throughout the study. Anthropometric measures of weight and height and demographic data and information of health-related behaviors were obtained prior to the test session. During each test walks, two physiotherapists were positioned by each cone and, blinded from the other physiotherapist’s counting, counted the steps with a click-counter. The testers were the same for all participants.

### Statistical analysis

Normal distributions of continuous data (steps, age, height, body mass index, meters walked in 6 min, and self-paced speed) were evaluated by quantile-quantile plots and histograms of the standardised residuals. Normally distributed continuous data were summarised by means and 95% confidence intervals. Continuous data without a normal distribution were summarised by medians and interquartile ranges. Categorical data were summarised with frequencies and percentage of the total score. The average of the visually counted steps from tester A and tester B was defined as the actual steps taken and hence the criterion. For every participant, four measures for each type of PAM were taken (left hip, right hip, left wrist, and right wrist). The frequency of excluded data points was reported and evaluated between groups with a Chi [2] test.

Interclass correlation coefficients (ICC) were calculated based on a two-way random effects analysis of variance model examining the absolute agreement of a single measure (ICC2,1) [21, 22]. We chose ICC2,1 as the raters were the same, and each participant was rated only once (average between the two testers). The model was chosen to examine the agreement between observed steps and the steps counted by the PAM. ICC (2,1) values of < 0.5, =0.5- <  0.75, =0.75- <  0.9, and ≥ 0.90 were interpreted as the PAM having, respectively, poor, moderate, good, and excellent criterion validity [21, 23]. Interclass correlation coefficients of mean difference in steps between observed steps and measured steps as well as percent measurement error were reported for 1) all participants, 2) participants without a rollator and 3) participants with a rollator. Our prior expectation was that each of the PAMs, would have at least moderate criterion validity for all participants (but with a low precision of the estimate because of the heterogeneity of the population), a good criterion validity for participants walking without a rollator and a poor criterion validity for participants walking with a rollator (as a previous study has shown that some PAMs have lower measurement properties among rollator users [12]). We expected a better criterion validity in participants without rollators because they were expected to walk faster and more similar to younger adults, compared with participants with rollators.

Visualisation of the absolute percentage measurement errors for each PAM was presented with a scatter plot and analysed with a generalised linear logit link model.

StataCorp. 2017. *Stata Statistical Software: Release 15*. College Station, TX: StataCorp LLC, was used for all statistical analyses and visualisations. An alpha level on 0.05 was considered the threshold for statistical significance.

## Results

**Fig. 1:**
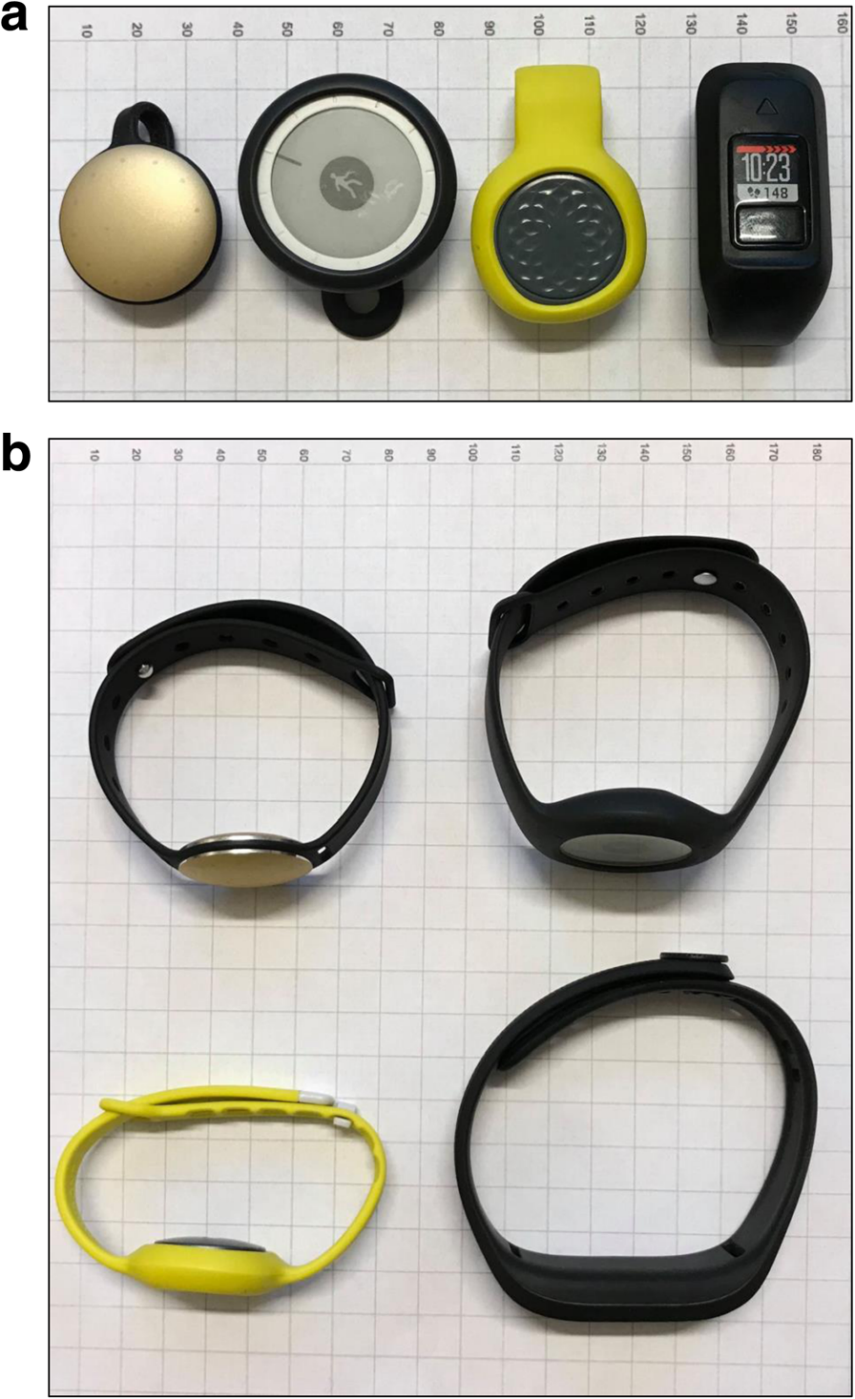
**a** and **b** From left to right: Misfit Shine, Nokia GO, Jawbone UP Move and Garmin Vivofit 3 on paper with 10-mm grid lines. Figure a shows the hip-worn physical activity monitors and below figure b shows the wrist-worn physical activity monitors

We identified four consumer-grade wearable PAMs available on the commercial market that met our eligibility criteria: Misfit Shine, Nokia GO, Jawbone UP Move and Garmin Vivofit 3. Below, Fig. 1 a and b show the four included monitors as they were used in this study. Only the Garmin Vivofit 3 included a regular display. The other monitors used light or illustrations to show how close the user is to the step goal of the day. Thus, the Garmin Vivofit 3 was the only monitor that could be operated without a smartphone device for this study.

A total of 103 older adults volunteered to participate in this study. Anthropometric, demographic data and information on health-related behaviour are presented in Table 1.

**Table 1:** Participants characteristics (*n* = 103)

Sex, male, n (%)	35 (34.0%)
Age, mean (95%CI)	81.3 years (79.8 to 82.8)
Height, mean (95%CI)	164.0 cm (162.2 to 165.9)
Body mass index, mean (95%CI)	28.0 kg/m [2] (27.0 to 29.0)
Self-paced meters walked in 6 min, mean (95%CI)	255.0 m (238.5 to 271.4)
Self-paced speed over the 6 min, mean (95%CI)	2.6 km/t (2.4 to 2.7)
Walking without aid, n (%)	52 (50.5%)
Walking with a cane, n (%)	15 (14.5%)
Walking with a rollator, n (%)	36 (35.0%)
Never smoked, n (%)	44 (42.7%)
Stopped smoking, n (%)	48 (46.6%)
Current smoker, n (%)	11 (10.7%)

Abbreviations: 95%CI: 95% Confidence interval; *IQR* Interquartile Range

Normal distributed continuous data: Age, Height, Body Mass Index, Meters walked in 6 min, Self-paced speed over the 6 min

### Deleted observations due to missing data and technical issues

The frequencies of excluded data points due to technical issues are listed in Table 2. We were unable to perform the necessary synchronization of the Nokia GO between each participant; thus, it was not possible to extract data for individual participants from the devices as the Nokia GO does not provide on the PAM itself the number of steps taken. Hence, the Nokia GO devices were excluded from the study. After April 1, 2018, an update to the Misfit iOS application, resulted in a malfunction in the synchronization between the iPod Touch and the Misfit monitors. As a result of this we had to excluded two of the Misfit monitors from that date. The remaining two monitors were positioned on the dominant side of the participants. In total, 103 data points were available for the Garmin and Jawbone monitors, 37 for the left-worn Misfit monitors and 99 for the right-worn Misfit monitors.

**Table 2:** A priori hypothesis for criterion validity, criterion validity, mean difference between measured steps and observed steps, and mean percentage measurement error for each physical activity monitor separately for each position

Position and type of monitor	A priori hypothesis ICC(2,1)	ICC(2,1) (95%CI)	Mean difference (95%CI)	Mean % measurement error (95%CI)
Hip-worn Misfit Shine, left				
All (34 measures)	0.5 to 0.75	**0.52 (0.21 to 0.73)**	−61.99 (− 104.50 to − 19.50)	−12.46% (− 21.09 to − 3.83)
Rollator (11 measures)	< 0.5	**0.56 (0.04 to 0.86)**	−73.00 (− 163.94 to 17.94)	−15.87% (− 35.30 to 3.55)
Without rollator (23 measures)	≥ 0.75	0.49 (0.13 to 0.75)	−56.71 (− 107.56 to − 5.88)	− 10.83% (− 20.76 to − 0.90)
Hip-worn Misfit Shine, right				
All (88 measures)	0.5 to 0.75	**0.64 (0.47 to 0.75)**	−48.35 (− 74.47 to − 22.24)	−8.75% (− 14.10 to − 3.40)
Rollator (31 measures)	< 0.5	0.44 (0.08 to 0.69)	− 110.19 (− 169.15 to − 51.24)	− 20.44% (− 31.87 to − 9.02)
Without rollator (57 measures)	≥ 0.75	**0.78 (0.66 to 0.87)**	−14.72 (− 36.11 to 6.65)	− 2.38% (− 7.39 to 2.61)
Hip-worn Garmin Vivofit 3, left				
All (100 measures)	0.5 to 0.75	**0.67 (0.53 to 0.78)**	−41.49 (− 64.21 to − 18.76)	− 9.74% (− 15.16 to − 4.33)
Rollator (36 measures)	< 0.5	**0.57 (0.19 to 0.78)**	−87.5 (− 131.23 to − 43.83)	−20.61% (− 31.02 to − 10.18)
Without rollator (64 measures)	≥ 0.75	0.71 (0.56 to 0.81)	−15.59 (− 39.88 to 8.71)	−3.63% (− 9.42 to 2.16)
Hip-worn Garmin Vivofit 3, right				
All (102 measures)	0.5 to 0.75	**0.80 (0,72 to 0,87)**	−22,61 (− 37.50 to − 7.72)	− 5.18% (− 9.01 to − 1.36)
Rollator (35 measures)	< 0.5	**0.74 (0.45 to 0.87)**	−44.11 (− 70.02 to − 18.21)	− 10.12% (− 16.60 to − 3.64)
Without rollator (67 measures)	≥ 0.75	**0.83 (0.73 to 0.89)**	−11.38 (− 29.46 to 6.70)	−2.61% (− 7.35 to 2.13)
Hip-worn Jawbone UP Move, left				
All (84 measures)	0.5 to 0.75	**0.61 (0.34 to 0.76)**	−63.75 (− 87.94 to − 39.56)	−13.11% (− 18.24 to − 7.98)
Rollator (23 measures)	< 0.5	0.40 (0.00 to 0.72)	− 101.65 (− 144.66 to − 58.64)	−19.21% (− 27.48 to − 10.94)
Without rollator (61 measures)	≥ 0.75	0.64 (0.44 to 0.78)	−49.45 (− 78.44 to − 20.48)	− 10.81% (− 17.19 to − 4.43)
Hip-worn Jawbone UP Move, right				
All (92 measures)	0.5 to 0.75	0.47 (0.21 to 0.65)	−85.79 (− 116.65 to − 54.95)	−16.57% (− 23.02 to − 10.12)
Rollator (31 measures)	< 0.5	0.24 (0.00 to 0.54)	− 193.83 (− 258.89 to − 128.78)	−38.28% (− 51.73 to − 24.84)
Without rollator (61 measures)	≥ 0.75	0.68 (0.51 to 0.80)	− 30.89 (− 54.96 to − 6.83)	−5.53% (− 10.86 to − 0.20)
Wrist-worn Misfit Shine, left				
All (36 measures)	0.5 to 0.75	0.18 (0.00 to 0.46)	−238.43 (−313.06 to − 163.81)	−44.21% (− 57.66 to − 30.78)
Rollator (12 measures)	< 0.5	0.00 (0.00 to 0.07)	− 486.5 (− 568.45 to − 404.55)	−91.03% (− 95.79 to − 86.27)
Without rollator (24 measures)	≥ 0.75	0.37 (0.00 to 0.68)	− 114.40 (− 170.90 to − 57.89)	− 20.80% (− 31.65 to − 9.96)
Wrist-worn Misfit Shine, right				
All (88 measures)	0.5 to 0.75	0.23 (0.00 to 0.47)	− 220.38 (− 266.13 to −174.64)	− 41.91% (− 50.49 to − 33.34)
Rollator (30 measures)	< 0.5	0.02 (0.00 to 0.09)	− 462.83 (− 518.31 to − 407.34)	−89.03% (− 97.04 to − 81.02)
Without rollator (58 measures)	≥ 0.75	0.55 (0.10 to 0.77)	− 94.97 (− 124.91 to − 65.03)	− 17.55% (− 23.39 to − 11.71)
Wrist-worn Garmin Vivofit 3, left				
All (88 measures)	0.5 to 0.75	0.31 (0.06 to 0.52)	− 139.71 (−186.39 to − 93.05)	− 27.17% (− 36.14 to − 18.20)
Rollator (22 measures)	< 0.5	0.00 (0.00 to 0.08)	− 455.78 (− 524.28 to − 387.27)	−88.31% (− 98.97 to − 77.67)
Without rollator (66 measures)	≥ 0.75	0.67 (0.51 to 0.79)	−34.36 (− 61.45 to − 7.28)	−6.79% (− 12.43 to −1.15)
Wrist-worn Garmin Vivofit 3, right				
All (89 measures)	0.5 to 0.75	0.33 (0.08 to 0.53)	− 132.98 (− 179.05 to − 86.91)	− 26.47% (− 35.62 to − 17.33)
Rollator (23 measures)	< 0.5	0.01 (0.00 to 0.08)	− 455.17 (− 519.00 to − 391.34)	−88.98% (− 98.99 to − 78.96)
Without rollator (66 measures)	≥ 0.75	**0.76 (0.63 to 0.84)**	−20.70 (− 42.4786 to 1.069512)	−4.69% (− 10.21 to 0.82)
Wrist-worn Jawbone UP Move, left				
All (65 measures)	0.5 to 0.75	0.30 (0.03 to 0.52)	− 121.52 (−166.86 to − 76.19)	−21.87% (− 30.14 to − 13.61)
Rollator (7 measures)	< 0.5	0.01 (0.00 to 0.23)	− 480.86 (− 640.08 to − 321.64)	−84.97% (− 110.58 to − 59.38)
Without rollator (58 measures)	≥ 0.75	0.47 (0.18 to 0.68)	−78.15 (− 112.13 to − 44.18)	−14.25% (− 20.81 to − 7.70)
Wrist-worn Jawbone UP Move, right				
All (55 measures)	0.5 to 0.75	0.29 (0.02 to 0.53)	− 105.05 (−148.00 to − 62.11)	−18.89% (− 26.65 to − 11.13)
Rollator (3 measures)	< 0.5	0.00 (0.00 to 0.88)	− 386.0 (− 1157.00 to 384.91)	− 66.17% (− 195.37 to 63.01)
Without rollator (52 measures)	≥ 0.75	0.38 (0.06 to 0.61)	−88.84 (− 126.23 to − 51.46)	−16.16% (− 23.13 to − 9.19)

Criterion validity calculated using a two-way random, single measures, absolute agreement model end expressed as interclass correlation coefficient

Abbreviations; *ICC* Interclass Correlation Coefficient (bold equal fulfilling the a priori hypothesis), *MD* Mean Difference: 95% Confidence intervals

Measurement error in % were evaluated as being not normally distributed and are presented with median and interquartile range. ICC (2, 1) values that meet the a priori hypothesis are marked with bold

**Fig. 2:**
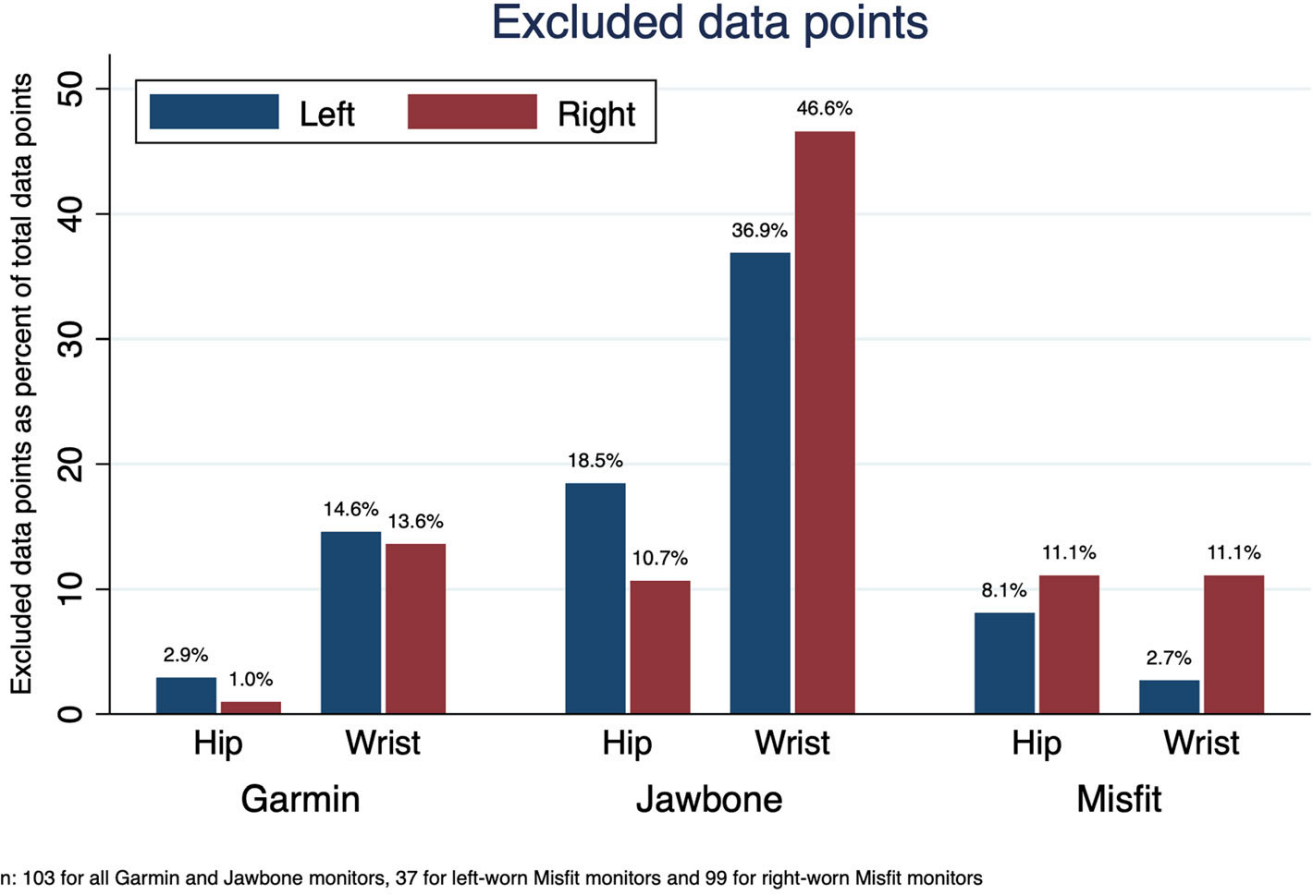
Excluded data points as a percentage of total data points sorted on brand and position. Higher percentage equals more excluded data. Chi [2]-tests revealed a significant difference between brands (*p* < 0.001) hip and wrist positions (p < 0.001) but not between left and right positions (*p* = 0.816)

Fig. 1 and 2 illustrates the percentage of excluded data points. In total, there were 175 excluded data points (16.0%), corresponding to 48 excluded hip measures (27.4%) and 127 excluded wrist measures (72.57%). A Chi [2] test revealed that wrist measures were more likely to be excluded (*p* <  0.001). In total, 8.0% of the Garmin Vivofit 3 measures, 28.2% of the Jawbone UP Move measures, and 9.6% of the Misfit Shine measures were excluded. A Chi [2] test revealed a significant between-group difference (p <  0.001). In total, 16.3% of the left-side measures and 15.7% of the right-side measures were excluded. A Chi [2] test revealed a no between-group difference (*p* = 0.816).

Table 2, reports results on criterion validity ICC (2,1), mean difference and percentage measurement error for all PAMs on all positions. For the hip-worn monitors, 10 out of 18 possible combinations (brand, left/right, and with or without rollator) fulfilled the a priori hypothesis of criterion validity. For the wrist-worn monitors, only one combination fulfilled the a priori hypothesis of criterion validity. The hip-worn Misfit Shine fulfilled four out of six possible combinations of criterion validity (left/right for all participants, participants with rollators and participants without rollators). The hip-worn Garmin Vivofit 3 fulfilled five out of six combinations for criterion validity. The hip-worn Jawbone UP Move fulfilled one out of six combinations for criterion validity. For the wrist-worn PAMs, no combination fulfilled the a priori hypothesis for criterion validity except the right-worn Garmin Vivofit 3 for participants with rollators. Good interrater reliability, ICC (2,1) was found between the two testers 0.88 (95% CI 0.83 to 0.92), with a mean difference on 4.42 steps 95% CI (− 6.10 to − 14.91), (103 measures).

### Measurement error

**Fig. 3:**
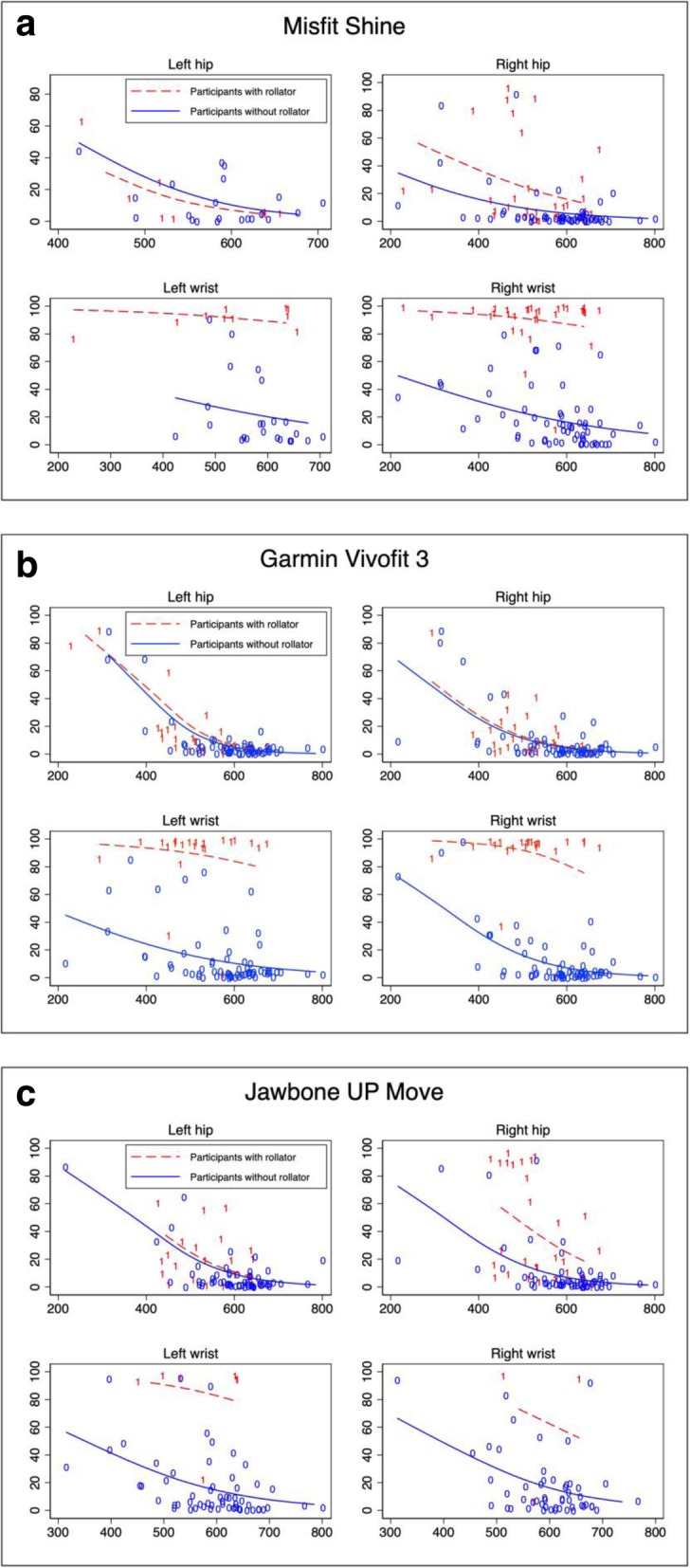
**a**, **b** and **c** Two-way scatter plots with logit link generalized linear models between absolute measurement error in % and observed steps (criterion) of Misfit Shine (a), Garmin Vivofit 3 (b) and Jawbone UP Move (c) physical activity monitors. Each figure includes results from the left hip, right hip, left wrist and right wrist. Red digits “1” and lines equal participants with rollators and blue digits “0” and lines equal participants without rollators. Y-axis represent absolute measurement error in % as a response to the x-axis which is number of observed steps

Fig. 3 a, b and c illustrates the relationship between measurement error in percent and observed steps. The logit link models reveal a negative slope for all PAMs in participants without rollators and for hip-worn monitors for participants with rollators. The models for wrist-worn monitors in participants with rollators differ from for the other models as the slope is more horizontal and has larger measurement error. There is no visual difference between any left and right measures.

## Discussion

The aim of this study was to investigate the criterion validity of four types of consumer-grade PAMs in older adults. The loss of data due to technical issues is more likely to happen with wrist-worn monitors. The Garmin Vivofit 3 showed the lowest frequency of lost data data-points and the Nokia GO was excluded from the study being incapable of synchronizing data. This means that we cannot rule out the Nokia GO as a PAM with acceptable measurement properties, as it might work very well with other devices. However, to be as transparent as possible, we chose to describe the Nokia GO with the same detail as the other PAMs. Hip-worn PAMs were superior to wrist-worn PAMs across all participants, participants without and with rollators in terms of criterion validity, absolute difference in steps, absolute measurement error in percentage and difference in steps.

Loss of data due to technical issues is often reported among consumer-grade PAMs [20]. In this study, none of the investigated PAMs was free from data loss but some of the PAMs were clearly more affected by this problem than others. Excluding lost data and zero counts will affect the criterion validity and cause a systematically higher interclass correlation compared to analysis with included zero counts. The interpretation of the ICC (2,1) value cannot stand alone and when evaluating the measurement properties of a PAM, results on data loss should be interpreted as well. Fig. 2 illustrates the problem in each brand, position and body side. The Garmin Vivofit 3 monitor and the Misfit Shine monitor had the lowest affection of data loss, but we had to exclude two of the Misfit monitors halfway, reducing the precision of our results. It also illustrates that wrist measures were more likely to be excluded, as many of the measures did not count when participants were using a rollator, and similarly, that there was no difference in exclusion of data between left and right-side measures.

The logit link models from Fig. 3 illustrate the relationship between measurement error and observed steps among participants with and without rollators. For the hip-worn PAMs among all participants and for wrist-worn PAMs among participants without rollators, the relationship was similar. In line with several other studies of consumer-grade PAMs in older adults, we found a higher accuracy in faster walking older adults [17, 19, 20, 24]. As described in the introduction, walkers with assistive devices are more likely to have alternative gait pattern compared to walkers using no assistive device. For participants using a rollator, the horizontal logit link models showed close to 100% absolute measurement error in wrist-worn PAMs indicating lack of arm movement among rollator users.

In terms of statistical methods, we chose to analyse the primary outcome using the two-way random effects model with absolute agreement and single measures, ICC (2, 1). In this model, each tester measures each participant, and testers are considered representative of a larger population of testers. Previously studies have either used Pearson correlation coefficients [19], unspecified ICC [17, 18] or ICC (2, 1) [12, 14, 15]. Agreement between two continuous outcomes should be reported using ICC values [25], and future studies should as a minimum report the specific sub-type of ICC as well as difference (percentage or mean) allowing the results to be compared between studies.

The criterion represents the actual true number of steps taken. When visually counting the steps, we avoided technical solutions of counting steps for the criterion. Other papers have often used research-grade accelerometers to validate consumer-grade PAMs [12, 14–19] which is the best option for free-living conditions. However, strictly for walking, the validity of research-grade PAMs can be questioned in this population as consumer-grade PAMs have been reported to have greater validity in trials comparing them to research-grade PAMs against visually counted steps [15]. With complex gait patterns in populations containing participants with and without walking aids the visually counted number of steps must serve as the most valid criterion, which was why we chose this method and in contrast to other studies with visual counts, we tried to reduce counting bias by having two testers instead of only one [15]. To exclude all error from the criterion, we could have combined more testers but it was not possible in this setting. However, all methods will have flaws and since there was no significant difference between the counts of the testers, we should be able to trust the average as a true criterion.

This study holds several limitations in the interpretation of the results. Firstly, the results are only generalisable to self-paced indoor walking in older adults. A study by Grant et al. reported large differences between counts from some research-grade PAMs in indoor treadmill walking and outdoor walking, but only in the slowest walking speeds [26]. To our knowledge, no published similar comparison has been made in free walking and using consumer-grade PAMs, but this highlights the lack of evidence in this area. Furthermore, the approach used for this study was general and covers only cyclic gait. The outcome of interest was step count when walking and did not include specific movements such as turning or squatting. Thus, our results only cover validity in cyclic gait and these results cannot be generalised and should not be extrapolated to conclude upon accelerometery vector counts in more specific movements. To investigate this, the raw data from the consumer-grade PAMs must be available for researchers, and until then, consumer-grade PAMs still remain as “black boxes” with hidden filtering software.

Secondly, we cannot rule out the possibility of existing PAMs, fulfilling our inclusion criteria that we were not aware of. We searched the literature and the web pages of all the major brands for relevant PAMs, but in the end our results do not apply other PAMs than the four devices we included in this study.

Another limitation is the possible systematic error in our dataset due to different track lengths (15 or 30 m) in the five different test locations. We cannot control for this in our model as it was not noted. Furthermore, the opportunity for participants to rest during the six minutes, could also produce a bias as resting in a chair, leaning against the wall or merely standing could be measured differently by the PAMs. As we do not have the data to distinguish between and investigate these possible types of error further, we cannot investigate the magnitude or direction of this possible systematic error.

Lastly, this study did not investigate intra-model test-retest reliability, but in terms of methodology, this type of reliability is almost impossible to investigate in PAMs as the same walking pattern and hence the individual participant cannot be repeated completely. However, despite the within-individual variation in gait pattern, it would be beneficial to do an intra-person reliability test-retest study of physical activity monitors in the future.

This study also holds several strengths. To our knowledge, this study includes the largest sample size reported in the literature on validation of consumer-grade PAMs in older adults. Furthermore, this is the first study that reports results on three different models, in two different positions, and it is with another study the only one with results on subgroups using different assistive devices [12, 14–20]. The latter makes the results of this study relevant to all populations that include both older adults with and without assistive devices. The results of this validation study are easily interpreted and the conclusion should be easily transferred to research groups planning to conduct clinical studies with PAMs as an outcome measure in older adults with different use of assistive devices.

Consumer-grade PAMs can potentially replace more expensive research-grade PAMs in situations where the level of physical activity should be measured or enhanced in older adults [20]. PAMs need not have excellent validity and reliability to serve as facilitators, but if they are to be used in research settings and serve as outcome measurements, validity and reliability are key to trust the results. Clinical studies that use consumer-grade PAMs as outcome measures should use hip-worn devices, especially if the target group holds older adults with and without rollators.

## Conclusion

Three of the four included consumer-grade PAMs were analysed and they showed varying measurement properties related to criterion validity among older adults performing a self-paced walking task. Our results show that wrist-worn PAMs cannot measure the number of steps in a population of older adults using rollators. The hip-worn PAMs were not significantly different in terms of measurement error or criterion validity, but when selecting a PAM for a clinical study, investigators should consider both the criterion validity and the rate of data loss as this also varied between monitors.

## Data Availability

The datasets used and/or analyzed during the current study are available from the corresponding author on reasonable request.

## Abbreviations

ICC2,1: Interclass correlation coefficient 2,1
PAMs: Physical activity monitors
